# Characterization of GABAergic marker expression in prefrontal cortex in dexamethasone induced depression/anxiety model

**DOI:** 10.3389/fendo.2024.1433026

**Published:** 2024-10-17

**Authors:** Ling Hu, Ming-Jing Qiu, Wen-Juan Fan, Wan-Er Wang, Shao-Hao Liu, Xiao-Qi Liu, Shi-Wei Liu, Ze-Jin Shen, Ya-Fei Zheng, Guang-Chao Liu, Zi-Yi Jia, Xiao-Qing Wang, Na Fang

**Affiliations:** ^1^ Department of Pathogen Biology, School of Basic Medical Sciences, Henan University, Kaifeng, China; ^2^ State Key Laboratory of Medical Neurobiology and MOE Frontiers Center for Brain Science, Institutes of Brain Science, Fudan University, Shanghai, China; ^3^ Neurological Department of Tongji Hospital, School of Medicine, Tongji University, Shanghai, China; ^4^ Luohe Medical College, Henan Province Engineering Research Center of Nutrition and Health, Luohe, China; ^5^ Henan Provincial Engineering Center for Tumor Molecular Medicine, Kaifeng Key Laboratory of Cell Signal Transduction, Henan University, Kaifeng, China

**Keywords:** chronic dexamethasone stress, prefrontal cortex, GAD67, reelin, GABAergic interneurons, depression and anxiety, chronic restraint stress

## Abstract

**Background:**

The pivotal responsibility of GABAergic interneurons is inhibitory neurotransmission; in this way, their significance lies in regulating the maintenance of excitation/inhibition (E/I) balance in cortical circuits. An abundance of glucocorticoids (GCs) exposure results in a disorder of GABAergic interneurons in the prefrontal cortex (PFC); the relationship between this status and an enhanced vulnerability to neuropsychiatric ailments, like depression and anxiety, has been identified, but this connection is still poorly understood because systematic and comprehensive research is lacking. Here, we aim to investigate the impact of dexamethasone (DEX, a GC receptor agonist) on GABAergic interneurons in the PFC of eight-week-old adult male mice.

**Methods:**

A double-blind study was conducted where thirty-two mice were treated subcutaneously either saline or DEX (0.2 mg/10 ml per kg of body weight) dissolved in saline daily for 21 days. Weight measurements were taken at five-day intervals to assess the emotional changes in mice as well as the response to DEX treatment. Following the 21-day regimen of DEX injections, mice underwent examinations for depression/anxiety-like behaviours and GABAergic marker expression in PFC.

**Results:**

In a depression/anxiety model generated by chronic DEX treatment, we found that our DEX procedure did trigger depression/anxiety-like behaviors in mice. Furthermore, DEX treatment reduced the expression levels of a GABA-synthesizing enzyme (GAD67), Reelin, calcium-binding proteins (parvalbumin and calretinin) and neuropeptides co-expressed in GABAergic neurons (somatostatin, neuropeptide Y and vasoactive intestinal peptide) in the PFC were reduced after 21 days of DEX treatment; these reductions were accompanied by decreases in brain size and cerebral cortex thickness.

**Conclusion:**

Our results indicate that a reduction in the number of GABAergic interneurons may result in deficiencies in cortical inhibitory neurotransmission, potentially causing an E/I imbalance in the PFC; this insight suggests a potential breakthrough strategy for the treatment of depression and anxiety.

## 1 Introduction

The etiology of numerous mental health conditions, particularly depression and anxiety, is closely linked to stress, underscoring stress as a key determinant in the progression of these disorders in humans and animals (1). Depression, also known as depressive disorder, constitutes a sophisticated mental health condition that exerts its influence on about 350 million individuals worldwide (2). With the accelerating pace of life, recent years have witnessed a notable uptrend in prevalence, and it is predicted to have the highest burden of disease in the future. Depression is divided into three severity categories: mild, moderate, and severe. The key clinical symptoms include low mood, anhedonia (the inability to experience pleasure from natural rewards), irritability, difficulty concentrating, abnormal appetite and sleep, lack of energy, inability to enjoy life, and suicidal thoughts, especially in individuals suffering from major depressive disorder (3). Furthermore, depression can increase the risk of heart disease, cerebrovascular disease, type 2 diabetes and other causes of death (4). The psychiatric condition of anxiety is typified by unwarranted and overwhelming concerns, physical agitation, and tiredness (5). Various subtypes of anxiety exist, such as general anxiety disorder, panic disorder, social anxiety, agoraphobia, posttraumatic stress disorder, and obsessive-compulsive disorder (6–9). At present, many types of antidepressants and anxiolytics that are used in the clinic are able to effectively help many patients; nonetheless, a high percentage of patients have delayed responses or no response to treatment and develop certain side effects, suggesting that the current pathophysiological understanding of depression/anxiety is still very weak. In particular, the methods and techniques for observing intracranial lesions are limited; researchers do not yet have the means to explain a series of complex symptoms, let alone resolve the weaknesses of current drug treatments for depression/anxiety (10). Given this situation, rodent models may be an important resource for exploring the fundamental mechanism of depression/anxiety, potentially advancing the research on core symptoms and aiding in the development of targeted treatments (11, 12).

Investigations utilizing rodent models have revealed that chronic stress or repeated intraperitoneal injection of glucocorticoids causes similar mental symptoms to depression/anxiety (13, 14). One key element of the stress response is the activation of the hypothalamic–pituitary–adrenal (HPA) axis accompanied by elevated levels of circulating glucocorticoids to offer optimal physiological assistance in the acute phase of the fight-or-flight response (15). The primary discoveries reported in research consistently demonstrate an upsurge in HPA axis activity in depression, accompanied by hypercortisolemia and reduced inhibitory feedback (16). It has been consistently proven through evidence that dysfunction of the glucocorticoid receptor occurs in cases of anxiety disorders (17). These results propose that the dysregulation of the HPA axis stems from an imbalance between glucocorticoids and glucocorticoid receptors (GRs) (18).

Stress hormone-related receptors such as GR are prominently expressed in the prefrontal cortex (PFC), which is a crucial brain region for the stress response and the core brain region through which chronic stress damages the emotional functions of the brain (19). The primate PFC, located in the cerebral cortex, is composed of the medial prefrontal cortex (mPFC), which is further subdivided into a ventral component (vmPFC) and a dorsal component (dmPFC). In particular, areas 25 and 32 of the vmPFC and area 24 of the dmPFC are homologous to the rodent infralimbic cortex (IL), prelimbic cortex (PL) and anterior cingulate cortex (ACC), respectively (20, 21). Therefore, the alterations in both structure and function of the prefrontal cortex (PFC) in rodents exposed to prolonged stress may be a critical link at which the mechanism of depression/anxiety could be interrupted by novel treatments.

Existing studies have suggested that the PFC contains rich inhibitory neurons γ-GABAergic neurons, mainly release the neurotransmitter of γ-aminobutyric acid (GABA) via two enzyme isoforms (GAD65 and GAD67) (22). GABAergic cells can be classified into three types: the somatostatin (SST) group, the parvalbumin (PV) group and the ionotropic serotonin 3A receptor (5HT3AR) group (23, 24). Each category comprises various subgroups, with the potential for commonalities. The SST group, constituting approximately 30% of GABAergic neurons, contains neuronal nitric oxide synthase (nNOS) and calcium-binding proteins (calbindin and calretinin) and colocalizes entirely with neuropeptide Y (NPY). Around 40% of GABAergic neurons are categorized under the PV group, which consists of fast-spiking basket cells and chandelier cells. The group of 5HT3AR, constituting approximately 30% of the total interneuronal population, expresses Reelin; some also express the neuropeptide vasoactive intestinal peptide (VIP), while others do not. Delving into the functions of these cell subtypes has proven beneficial in pinpointing molecular susceptibilities encompassing depression/anxiety and other stress-related disorders.

Chronic stress-triggered emotional disorders like anxiety and depression arise from disruptions in the balance between the excitatory glutamatergic system and the inhibitory GABAergic system (25–29). It is worth noting that the changes in GABAergic neurons after stress, especially excessive glucocorticoids, remain controversial (27, 30, 31). Recent studies have identified significant increases in calbindin, GAD65 and GAD67 but failed to demonstrate differences in CR and PV levels without behavioral changes in response to chronic glucocorticoid exposure (31). Increasing evidence has suggested that a reduced number of PV-immunopositive neurons is accompanied by working memory deficits and anxiety- and depression-like behavior in DEX-treated rats (32). In addition, there remain other subgroups (e.g., neuropeptide Y and Reelin) for which data are missing (33, 34). Therefore, a primary objective of this study was to explore the impact of elevated glucocorticoid levels on specific subtypes of GABAergic inhibitory neurons.

The current investigation utilized a chronic DEX model, which has been authenticated in our lab, to duplicate various behavioral and cellular aspects of depression (35). Anxiety-like behaviors were also observed in DEX-treated mice, along with reduced brain size and pronounced thinning of the cerebral cortex. Strikingly, we found evidence that excessive glucocorticoids impaired the GABAergic system in the PFC, which changed the excitation/inhibition ratio. This abnormality of GABAergic neurons may be caused by dysfunction of the HPA axis. This could provide fresh perspectives for a more thorough understanding of the biology of these refractory disorders and, most importantly, for application in prompt identification and treatment.

## 2 Materials and methods

### 2.1 Experimental animals

All experimental procedures underwent thorough review and were granted approval by the Animal Committee of the Department of Laboratory Science, Henan University, China. Male C57BL/6J adult mice were group-housed in sets of 5 within an enclosure with a 12-hour light/dark regime (lights switched on at 7:00 a.m.), maintaining a controlled environment of 22 ± 2°C temperature and 50 ± 10% humidity, and were supplied with standard nourishment and water ad libitum.

### 2.2 Chronic DEX treatment

A double-blind study was conducted where thirty-two mice were treated subcutaneously either saline or DEX (0.2 mg/10 ml per kg of body weight; HY-14648, MedChemExpress, NJ) dissolved in saline daily for 21 days. Weight measurements were taken at five-day intervals to assess the emotional changes in mice as well as the response to DEX treatment. Following the 21-day regimen of DEX injections, mice underwent examinations for depression and anxiety-like behaviors. The study included 16 mice in each group - control and DEX. Eight mice from each group were designated for behavioral testing, while the remaining eight were used for anatomical and tissue staining analyses.

### 2.3 Behavioral tests

Following a 21-day induction of DEX, on day 22, male mice from the Control group (n=8) and the DEX group (n=8) were subjected to various behavioral assessments, comprising the sucrose preference test (SPT), tail suspension test (TST), forced swim test (FST), light-dark box (LDB) test, and open-field test (OFT) as part of a double-blind study design.

#### 2.3.1 Sucrose preference test

Depressive-like behavior was assessed through the SPT on the 22nd day for male mice allocated to the Control group (n=8) and the DEX group (n=8). Prior to the experiment, animals were individually housed for 3-7 days to minimize the influence of social factors on the experimental outcomes. Additionally, to familiarize the animals with the sweet substance, sucrose habituation training was conducted by providing them with a certain concentration of sucrose solution (e.g., 1% sucrose) to adapt to the taste. The sucrose solution used in the experiment typically ranged from 1% to 2% sucrose solution. To ensure the reliability of the experimental results, the consistency of the sucrose solution concentration was maintained throughout the experiment. The experiment commenced with the administration of sucrose solution and plain water to the animals simultaneously. The consumption of both liquids was periodically measured within a specific time frame (e.g., 24 hours). The calculation method for the Sucrose Preference Index (SPI) involved determining the post-experiment sucrose preference index, which is the ratio of sucrose intake to total intake (sucrose + plain water) (35).

#### 2.3.2 Tail suspension test

The TST was carried out on the 23rd day for the mice assigned to the Control group (n=8) and the DEX group (n=8). At the initiation of the experiment, animals were gently removed from their cages and their tails were promptly immobilized to prevent unnecessary stress and arousal. Specifically, medical tape was applied 1 cm away from the tail tip, following which the animals’ tails were suspended on the apparatus hanger. The height from the tail tip to the ground was approximately 30 cm, positioning the mice in a head-stand stance. The camera lens was adjusted in advance and centered on the mice’s bodies to ensure a comprehensive view of the recording. The experiment required a 6-minute video recording, capturing the immobility periods of the animals in the initial 2 minutes and the subsequent 4 minutes. Post-recording session, the mice were cautiously freed from the medical tape by peeling them off and then returned to their original cages with proper documentation. Following the completion of all animal tests, the apparatus was promptly cleaned of feces and urine (36).

#### 2.3.3 Forced swim test

On the 24th day, mice in the Control group (n=8) and DEX group (n=8) underwent the FST test (37). Prior to the experiment, the water temperature inside the testing apparatus was adjusted to a range of 23 to 25°C. The water depth was adjusted based on the animals’ body weight, ensuring a certain distance between the animals’ tails and the bottom of the testing apparatus. Each pair of animals was separated by an opaque barrier. The swimming time for the mice was set at 6 minutes, with immobility recorded during the subsequent 4 minutes (37).

#### 2.3.4 LDB test

On the 25th day, mice in the Control group (n=8) and DEX group (n=8) underwent the LDBT test. The open white rectangular chamber known as the dark-light box had dimensions of 40 × 40 × 33 cm^3^. It was designed with an entry point of 8 × 10 cm that led to a black compartment of the size 20 × 40 × 33 cm^3^. Our research included exposing the light chamber to an intensity of 500 lux. Every mouse was positioned inside the dark compartment, and the latency period was measured along with the number of visits to the light chamber within 5 minutes. Entry into the light chamber was confirmed only after the subject had crossed the threshold with all four limbs. Observation of their conduct was documented via digital camera, followed by analysis through the SYGNIS Tracking Program (38).

#### 2.3.5 Open field test

The OFT was administered to the Control group (n=8) and the DEX group (n=8) on the 26th day. The open-field apparatus comprised a square arena where the white floor was partitioned into nine 10 cm × 10 cm squares, enclosed by unbroken walls standing at 21 cm in height constructed of see-through acrylic glass. The experimental procedure extended over a period of 30 minutes. The activity monitoring software recorded the distance traveled in center zone (39).

In all, the evaluation of depression-like behaviors involved the utilization of the SPT, TST, and FST, while anxiety-like behaviors were assessed through the LDB test and OFT.

### 2.4 Nissl staining

Brains were harvested after DEX exposure and sectioned in a cryostat at 25 μm. Then, application of Nissl staining solution (Beyotime, C0117, Shanghai, China) to coronal cryosections occurred for a 5-minute duration at room temperature. Next, the samples underwent washing with 95% ethyl alcohol for a duration of 5 minutes followed by air drying. The sections were subsequently rinsed two times in xylene lasting 5 minutes each. Following application of neutral balsam sealing, the slides were examined using an optical microscope (Eclipse 80i, Nikon) by a blinded investigator. Cortical thickness was measured at the primary somatosensory cortex of forelimb (S1FL) region of the neocortex with the following coordinates: anteroposterior (AP) +0.74 mm, mediolateral (ML) +2.0 mm, dorsoventral (DV) -1.6 mm (40).

### 2.5 *In situ* hybridization

The procedure of *ISH* was carried out as detailed in earlier literature (41). To prepare probes for GAD67, Reelin, SST, VIP and NPY, total RNA was isolated from the cerebral cortex utilizing TRIzol as per the guidelines provided by the manufacturer (Qiagen). Each sample was reverse transcribed using PrimeScript™ RT reagent Kit with gDNA Eraser (Takara, RR047A), and a 400-600 bp complementary DNA (cDNA) fragment for each gene was subcloned into the pGEM-T vector (Promega, A362A), linearized for *in vitro* transcription and subjected to digoxigenin (DIG) RNA labelling (SP6/T7; Roche Diagnostics Ltd. UK). Detection and visualization of signals were conducted employing 5-bromo-4-chloro-3-indolyl phosphate (Boehringer Mannheim) and 4-nitroblue tetrazolium salt (BioRad) as substrates for alkaline phosphatase. The process of *ISH* was visualized through images obtained with a brightfield microscope (Eclipse 80i, Nikon).

### 2.6 Immunohistochemistry

Immunohistochemical staining was executed as per the described procedures in earlier research (42). Briefly, brain slices (25-μm thick) were harvested after behavioual testing. The primary antibodies utilized included: Parvalbumin (1:1000, rabbit, Swant, PV27) and Calretinin (1:1000, mouse, Swant, 6B3). After overnight incubation at 4°C with primary antibodies, the sections were subsequently incubated with biotin-conjugated secondary antibodies (Vector Laboratories) for 3 hours, followed by labeling with either Cy2-conjugated streptavidin or secondary antibodies conjugated to Alexa fluorochromes (Molecular Probes, Invitrogen). Slides were treated with 75% glycerol and images were captured utilizing an epifluorescence microscope (Eclipse 80i, Nikon) with the assistance of NIS-Elements F400 software.

### 2.7 Results analysis and quantification

Behavioral results were analyzed blinded to the experimental conditions (n = 8 for each group). Microscope images of the PFC were procured via microscopy, with a minimum of 8 individual samples examined in every group. The quantification of images from the PFC involved selecting sections spanning from approximately 4.20 mm to 1.20 mm referenced to Bregma. The number of total GABAergic-positive puncta (GAD67, Reelin, SST, VIP, NPY, PV and CR) and cortical thickness measurements were quantified through ImageJ software by one investigator blinded to the experimental conditions.

### 2.8 Statistical analysis

At least eight animals of each group were used for statistical analysis to determine significance in each experimental paradigm. Normal distribution was conducted on the collected data, followed by the application of Student’s t-test to compare the two groups. The statistical analysis was performed using GraphPad Prism software, version 8.0. The data was displayed as the average ± standard error of the mean. Significance was attributed to p values below 0.05.

## 3 Results

### 3.1 Effects of DEX treatment on depression/anxiety-like behaviors

**Figure 1:**
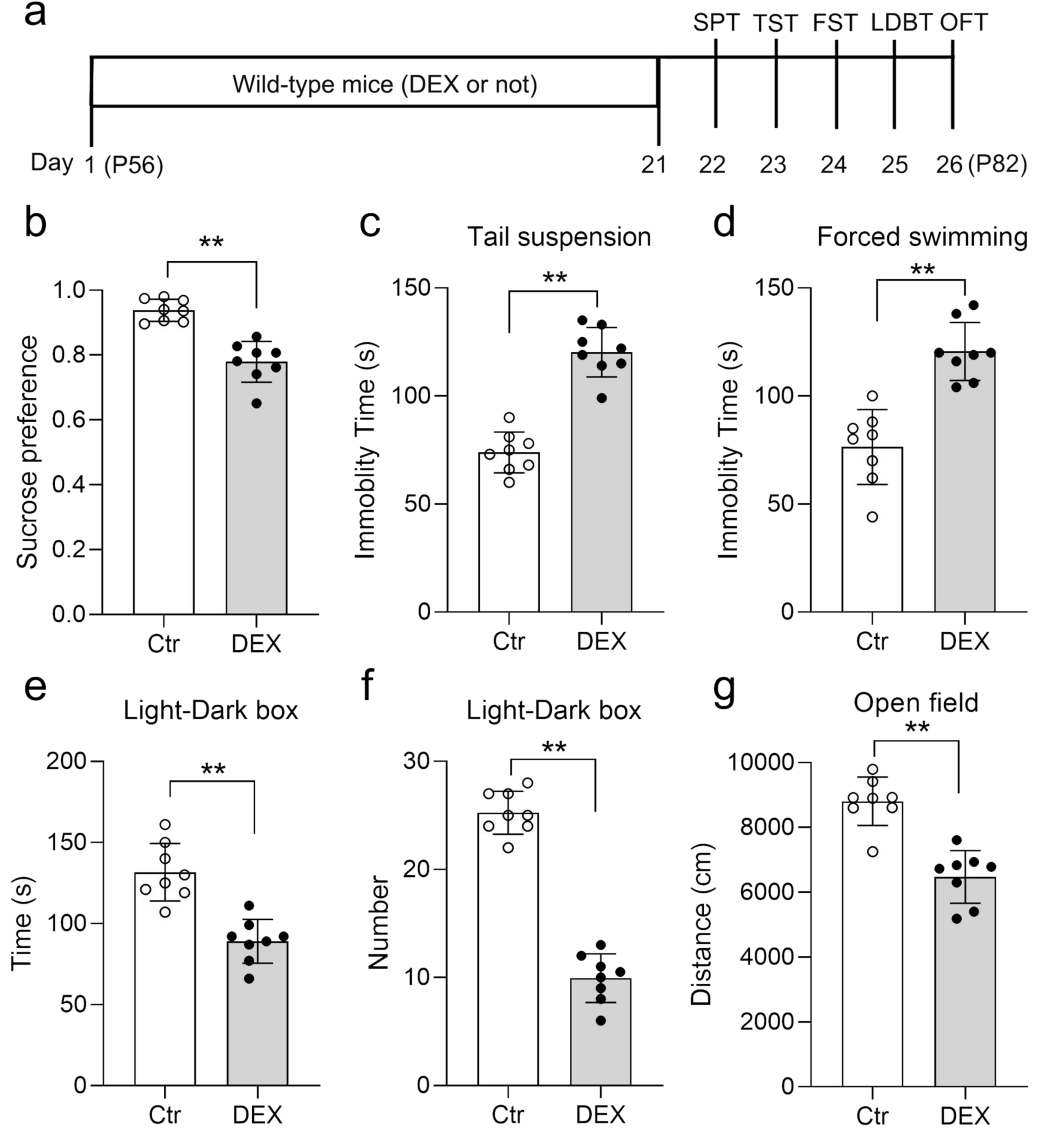
DEX treatment led to depression/anxiety-like behaviors. Diagram of the experimental design and timeline **(A)**. b–d The Dex treatment resulted in decreased sucrose preference **(B)** and increased immobility time in the TST **(C)**, and FST **(D)**. **(E, F)** The time spent in the light chamber **(E)** and the times mice shuttled between the two boxes **(F)** were decreased in the LDB test. **(G)** DEX resulted in a decreased distance in center zone of the OFT. n = 8 in each group and all the data are presented as mean ± SEM. Data were analyzed using Student’s t-tests **(B-G)**. **p < 0.01 (control mice versus DEX-treated mice). Dex, dexamethasone; FST, forced swim test; OFT, open-field test; SPT, sucrose preference test; TST, tail suspension test; LDBT, light-dark box test.

DEX functions as a synthetic agonist of glucocorticoids, and chronic DEX administration is commonly employed to trigger depression/anxiety-like behaviors in rodents (43–45). To validate our DEX protocol, a battery of behavioral examinations, encompassing the SPT, TST, FST, LDB test and OFT, were performed (**Figure 1A**). Our data showed that DEX treatment led to a diminishment in sucrose preference and a surge in the duration of immobility across both the TST and FST assessments (**Figures 1B-D**). As expected, the habit of exploring the light was weakened, including reduced immobility time in the light chamber and the times mice shuttled between the two boxes (**Figures 1E, F**). Locomotor activity was decreased in DEX-treated mice, as shown by the travelled distance in center zone relative to controls (**Figure 1G**). Taken together, our DEX procedure did trigger depression/anxiety-like behaviors in mice, according to the proof presented.

### 3.2 Effects of DEX treatment on the thickness of the cerebral cortex and brain size

**Figure 2:**
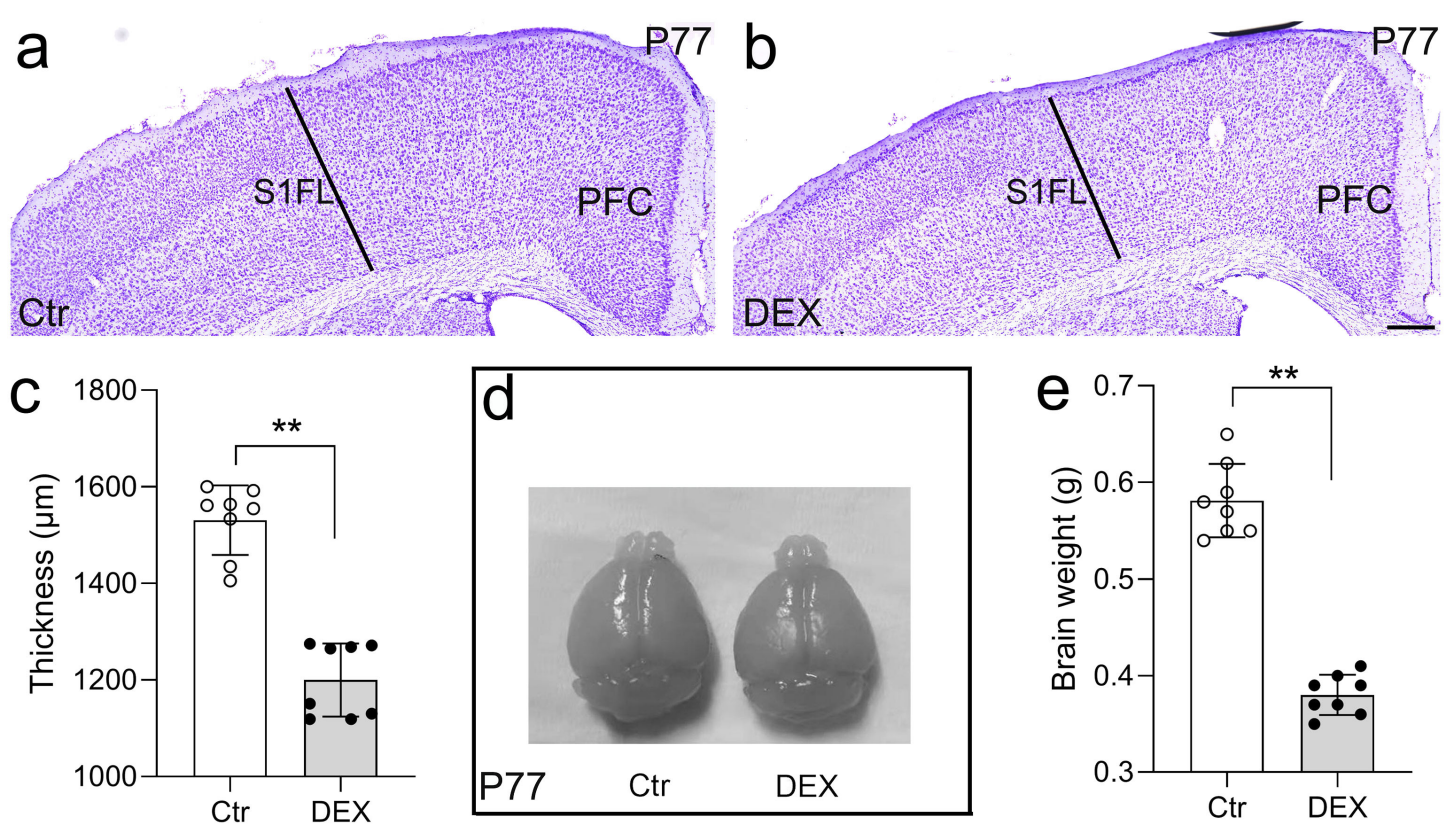
DEX led to reduced thickness of the cerebral cortex and brain size. **(A-C)** Nissl staining revealed a significantly reduced cortical thickness at the S1FL region of the neocortex in DEX-treated mice in comparison to control mice. n = 8 for each group. **(D, E)** The brain size of control and DEX-treated mice at P77; three weeks of DEX treatment induced a mild reduction in brain size. n = 8 in each group. All the data are presented as mean ± SEM. Data were analyzed using Student’s t-tests **(C, E)**. **p < 0.01 (control mice versus DEX-treated mice). DEX, dexamethasone; PFC, prefrontal cortex; S1FL, primary somatosensory cortex of forelimb.

Initial body weight measurements were taken at the onset of stress exposure for each individual mouse, with subsequent weight recordings conducted every five days until animals were sacrificed. In contrast, 21 days of DEX treatment prevented the increase in body weight (data not shown). We evaluated the gross cortical morphology of DEX-treated mice on day 77. When juxtaposed with control mice, the cortical thickness at the S1FL region of the neocortex in DEX-treated mice was notably decreased by Nissl staining (**Figure 2A-C**), consistent with the decreased brain size (**Figures 2D, E**) often found in major depressive disorder (46). We then surveyed the cortical thickness along the rostral-caudal axis and detected a general decrease in cortical thickness with no region-specific pattern after DEX exposure (**Supplementary Figure S1**). Collectively, the evidence suggests that our DEX protocol impairs cerebral cortex construction.

### 3.3 Effects of DEX treatment on GAD67 and Reelin in the PFC

**Figure 3:**
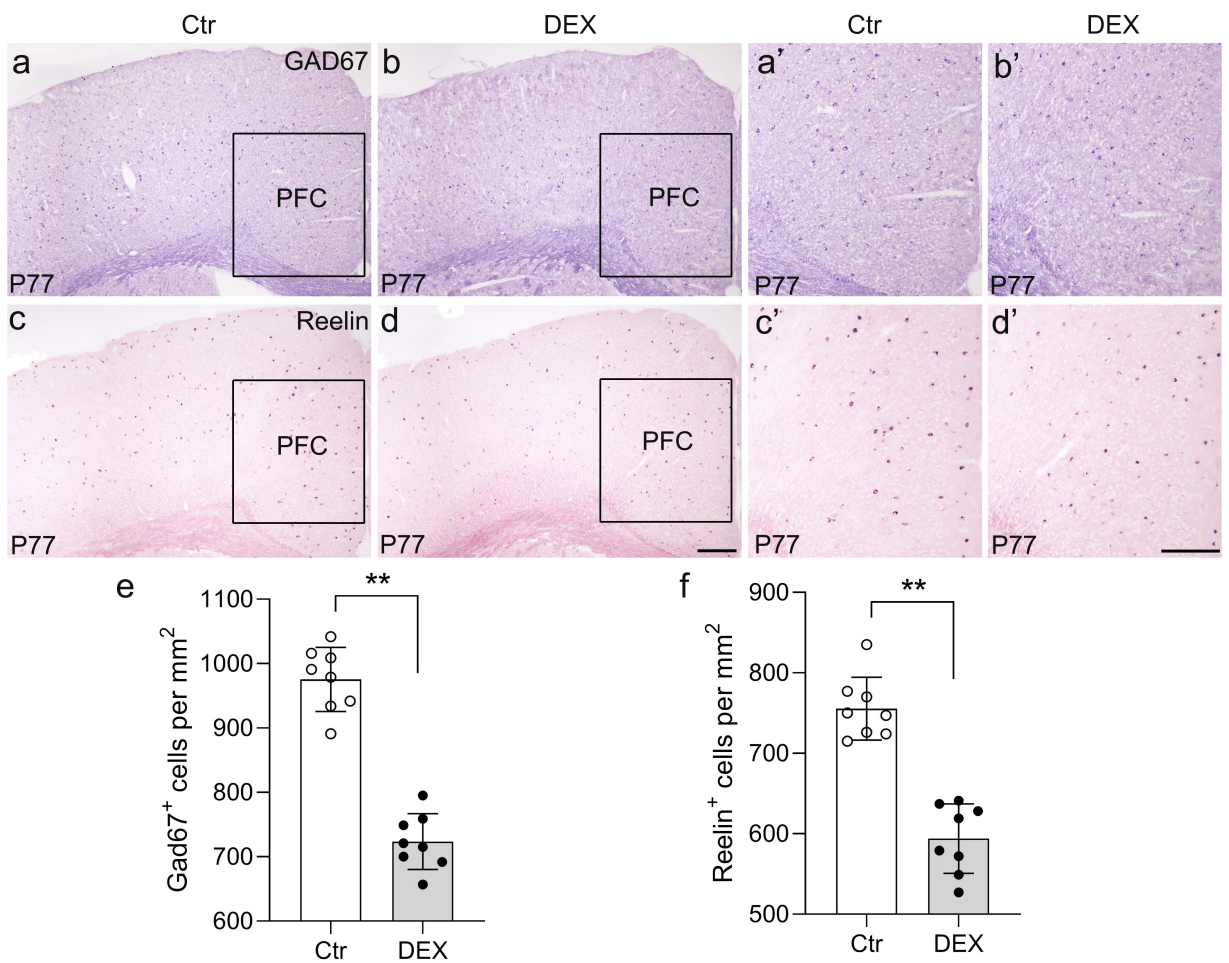
The DEX-treated mice had reduced GAD65 and Reelin expression. **(A-F)** Coronal sections from P77 brains stained with GAD65 and Reelin markers. **(A, B’)** GAD65 *in situ* hybridization showed decreased puncta in DEX-treated mice (**E**, *p*<0.01, **) compared with control mice. **(C, D’)** Reelin puncta were significantly reduced in DEX-treated mice, as shown by *in situ* hybridization of Reelin (f, *p*<0.01, **). The boxed area represents the PFC, and the areas in **(A-D)** are enlarged in **(A’-D’)**, respectively. Scale bar = 100 µm. n = 8 in each group. All the data are presented as mean ± SEM. Data were analyzed using Student’s t-tests. **p<0.01 (control mice versus DEX-treated mice). DEX, dexamethasone; PFC, prefrontal cortex.

To assay changes in the expression of a GABA-synthesizing enzyme marker (GAD67) and an important neurodevelopmental protein marker (Reelin, a glycoprotein preferentially secreted by cortical GABAergic interneurons) (47)in our depression/anxiety-like mouse model, the expression levels of GABAergic genes in the cerebral cortex were assessed through ISH analysis at P77. Then, our attention was directed towards the PFC, known as a highly susceptible area to stress and a crucial brain region associated with depression (48). Following 21 days of DEX treatment, the PFC exhibited a substantial reduction in GAD67 expression levels as compared to the control group (**Figures 3A, B, A’, B’, E**; n = 8/group, *p*<0.01). Similar to the change in GAD67, the number of Reelin^+^ neurons in the PFC was calculated, and the percentage revealed that Reelin levels were lower in the PFC following DEX exposure (**Figures 3C, D, C’, D’, F**; n = 8/group, *p*<0.01). Thus, our data demonstrate that the levels of gene expression for GAD67 and Reelin in the PFC are reduced by DEX exposure.

### 3.4 Effects of DEX treatment on calcium-binding proteins specific to GABAergic Interneurons in PFC

**Figure 4:**
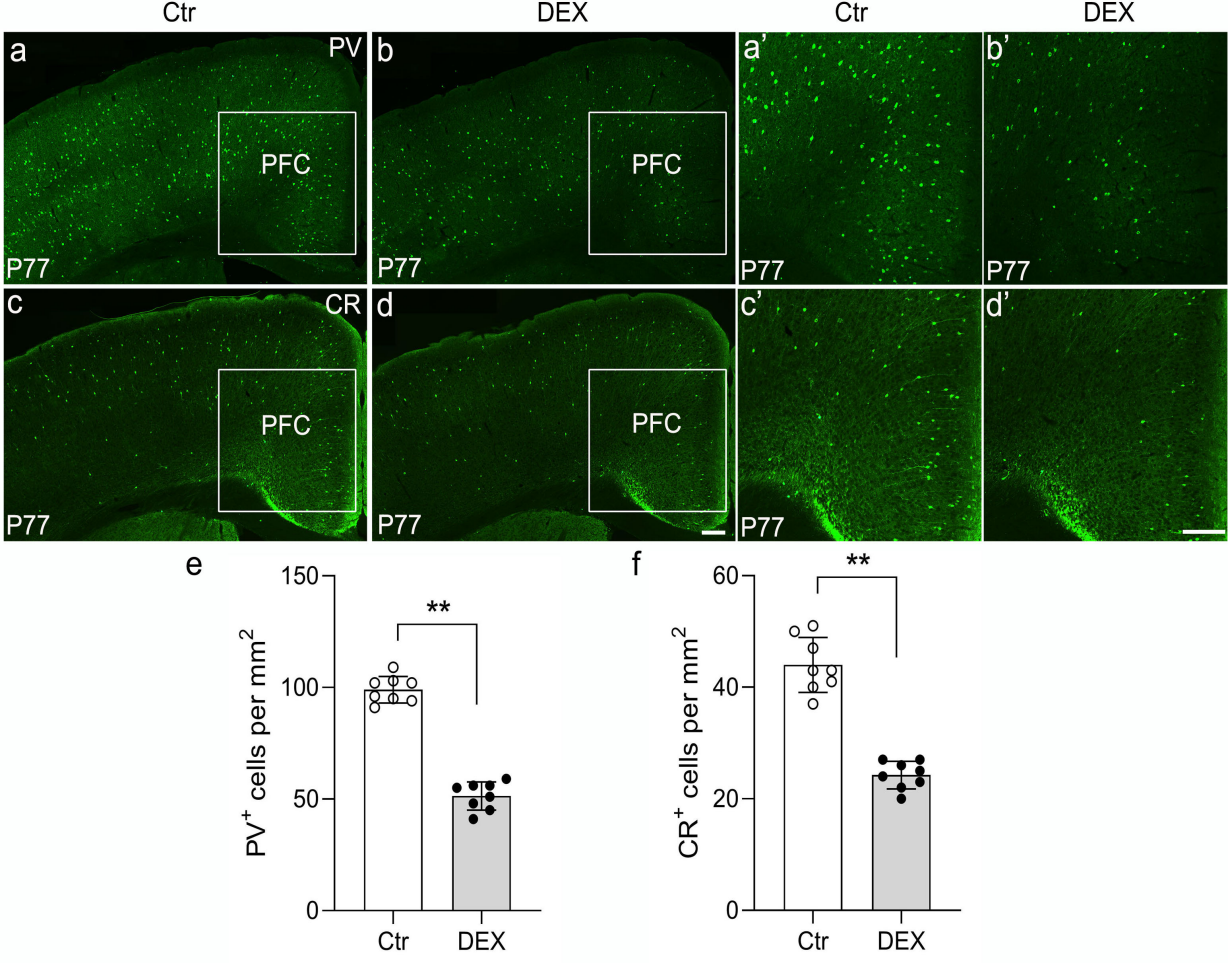
The DEX-treated mice had reduced PV and CR expression. **(A-F)** Representative images of PV and CR immunohistochemistry in the cortex of control and DEX-treated mice. **(A, B’)** Immunostaining of PV showed decreased puncta in the DEX-treated mice (**E**, p<0.01, **) compared with the control mice. **(C, D’)** Similar to the phenotypes observed in PV immunohistochemistry, a decreased number of CR^+^ cells was found in the PFC after stress (f, p<0.01, **). The boxed area represents the PFC, and the areas in **(A-D)** are enlarged in (A’-D’), respectively. Scale bar = 200 µm. n = 8 in each group. All the data are presented as mean ± SEM. Data were analyzed using Student’s t-tests. **p<0.01 (control mice versus DEX-treated mice). DEX, dexamethasone; PFC, prefrontal cortex.

To explore whether calcium-binding proteins co-expressed in GABAergic neurons are preferentially affected by DEX treatment, immunohistochemistry for PV and calretinin (CR) was performed in our depression/anxiety-like mouse model (**Figure 4**; representative images). Our representative images revealed that PV^+^ interneurons are drastically reduced in the PFC of stress-vulnerable mice in contrast with controls (**Figures 4A, B, A’, B’, E**; n = 8/group, *p*<0.01). Consistently, anti-CR staining indicated that reduction in the density of PV interneurons within the PFC was evident post DEX exposure (**Figures 4C, D, C’, D’, F**; n = 8/group, *p*<0.01). Thus, these results showed that PV puncta and CR puncta in the PFC are seriously reduced following DEX exposure.

### 3.5 Effects of DEX treatment on neuropeptides specific to GABAergic interneurons in PFC

**Figure 5:**
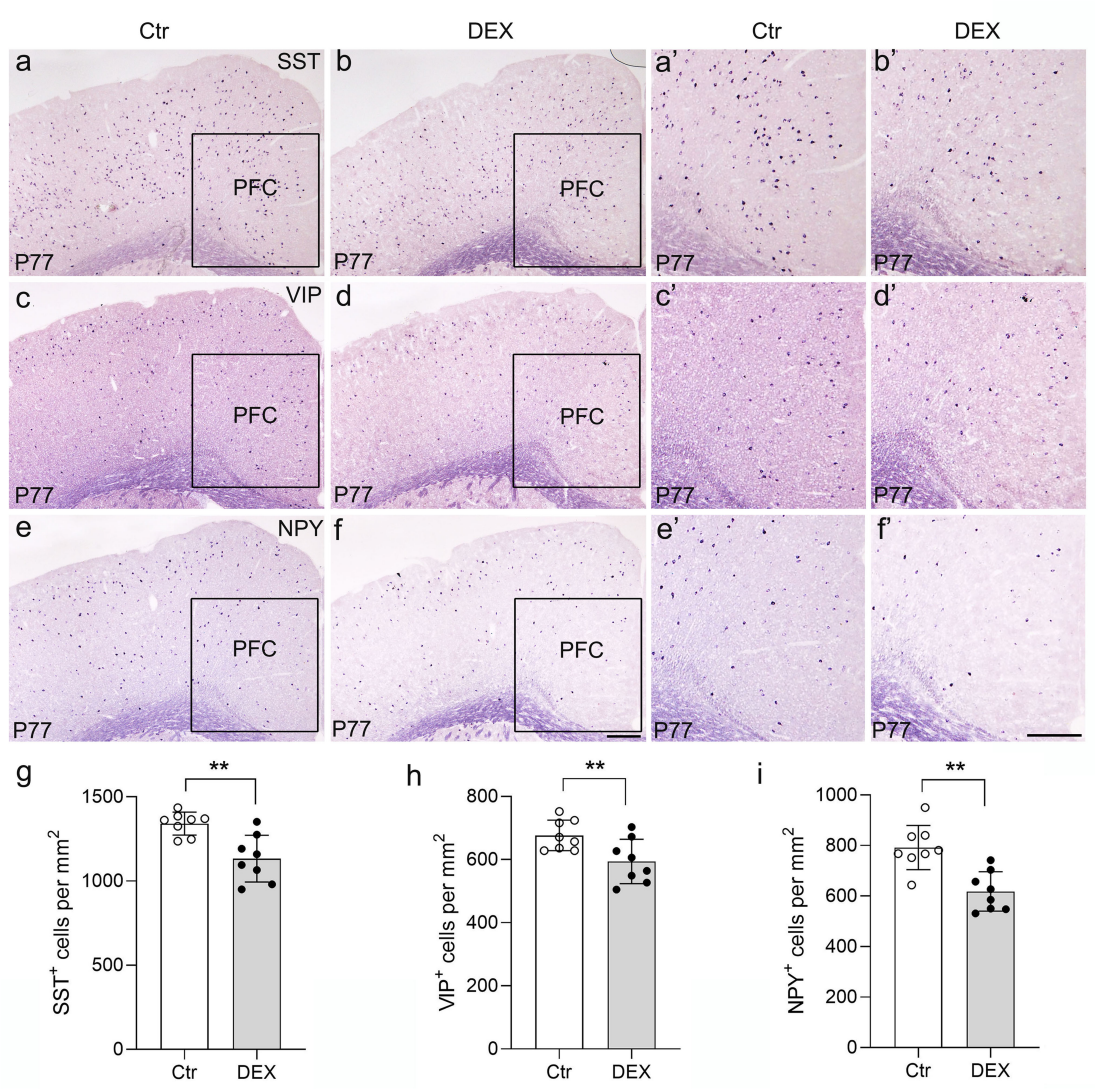
The DEX-treated mice had reduced neuropeptides specific to GABAergic interneurons. **(A-F)** Coronal sections from P77 brains stained with various neuropeptides specific to GABAergic interneuron markers. *In situ* hybridization of SST **(A, B’)**, VIP **(C, D’)** and NPY **(E, F’)** is illustrated. **(G-I)** Quantitative analysis showed that there was an approximately 10% decrease in the number of SST^+^ PFC regions after DEX exposure (**G**, p<0.01, **). Moreover, VIP and NPY expression detected in the PFC was significantly reduced in DEX-treated mice (**H-I**, p<0.01, **). The boxed area represents the PFC, and the areas in **(A-F)** are enlarged in **(A’-F’)**, respectively. Scale bar = 100 µm. n = 8 in each group. All the data are presented as mean ± SEM. Data were analyzed using Student’s t-tests. **p < 0.01 (control mice versus DEX-treated mice). Dex, dexamethasone. PFC, prefrontal cortex.

To delve deeper into the subtype-specific susceptibility of interneurons towards DEX administration, the gene expression levels of neuropeptides co-expressed in GABAergic interneurons were assessed using ISH analysis. In the PFC, we first carried out ISH of SST and found a dramatic decrease by quantitative analysis in DEX-treated mice (**Figures 5A, B, A’, B’, G**; n = 8/group, *p*<0.01). Following ISH procedures on VIP, it was evident that the quantity of VIP^+^ interneurons in the PFC notably diminished in mice treated with DEX (**Figures 5C, D, C’, D’, H**; n = 8/group, *p*<0.01). Eventually, we executed NPY staining and affirmed a marked decrease in the PFC of DEX-treated mice after careful quantification (**Figures 5E, F, E’, F’, I**; n = 8/group, *p*<0.01). These data strongly suggest that SST^+^, VIP^+^ and NPY^+^ interneurons are dramatically decreased following DEX exposure. In **Table 1**, we present the correlation between GABAergic neuron density and behavioral indicators in DEX-treated mice. The results show a remarkable association between the density of GABAergic neurons and depressive/anxiety-like behaviors, suggesting that the reduction in GABAergic neurons may be instrumental in the development of these behavioral abnormalities.

**Table 1:** Correlation between GABAergic neuron densityand behavioral performance in DEX-treated mice.

Behavior Cell counts	Sucrose Preference (%)	Tail Suspension (s)	Forced Swim (s)	Light-Dark box Time(s)	Light-Dark box Number	Open field Distance (cm)
Gad67^+^ Cells (per mm^2^)	0.947*	0.9627*	0.9348*	-0.7438*	-0.7606*	-0.7916*
Reelin^+^ Cells (per mm^2^)	0.8072*	0.8504*	0.8912*	-0.3439	-0.4552	-0.3647
PV^+^ Cells (per mm^2^)	0.836*	0.882*	0.8707*	-0.4738	-0.5141	-0.4967
CR^+^ Cells (per mm^2^)	0.725*	0.824*	0.8942*	-0.7426*	-0.7699*	-0.8446*
SST^+^ Cells (per mm^2^)	0.885*	0.8723*	0.921*	-0.6204	-0.6211	-0.7615*
VIP^+^ Cells (per mm^2^)	0.9377*	0.8433*	0.7863*	-0.3994	-0.4154	-0.4164
NPY^+^ Cells (per mm^2^)	0.9325*	0.9554*	0.8939*	-0.6284	-0.7678*	-0.5699

## 4 Discussion

The involvement of GABAergic signaling holds significance in the etiology of glucocorticoid-induced psychiatric disorders, including depression and anxiety disorders (49). We previously reported that our DEX protocol indeed induced depression-like behaviors (35). In this study, we demonstrated that DEX treatment leads to reduced exploration of the light chamber of the LDB test, along with deficient locomotor activity. In addition to alterations in anxiety-like behavior, the DEX-treated mice displayed damaged parts of the brain structure. Furthermore, DEX treatment reduced GABAergic markers in the PFC to varying degrees, which may underlie depression/anxiety-like behaviors.

Acting as critical stress response hormones, glucocorticoids facilitate stress coping. Chronic glucocorticoid exposure evokes neuronal cell damage and dendritic atrophy, reduces hippocampal neurogenesis and impairs synaptic plasticity. Glucocorticoids also alter expression and signaling of the neurotrophin, brain-derived neurotrophic factor (BDNF) (50). According to the research conducted by Lv and colleagues, it was discovered that the expression of BDNF was significantly upregulated in higher concentration of DEX treatment (39). Griesbach et al. found that restraint induced stress increases hippocampal Glucocorticoid Receptor, but decreased BDNF in fluid percussion injured rats (40). We used male mice of the same age in both the chronic restraint stress (CRS) protocol and the DEX protocol. However, it should be noted that there were differences in behavior between CRS-treated mice and DEX-treated mice. No significant changes were noted in locomotor activity in CRS-treated mice, as demonstrated by the distance traveled in center zone contrasted with the control mice, while the mice exhibited an anxiety-like behavioral phenotype after chronic DEX treatment. This discrepancy might be explained by the following factors. First, the concentration of glucocorticoids or expression level of BDNF was inconsistent in two models (51, 52). Maybe the concentration of DEX we used was higher. Second, DEX is a glucocorticoid agonist that may work via different mechanisms than glucocorticoids. Moreover, CRS may cause other hormonal changes, including corticosterone, growth hormones, prolactin, insulin, secretin etc (53). And it has been reported that chronic corticosterone administration induces negative valence and impairs positive valence behaviors in mice (54), and the administration of corticosterone was observed to influence depression-like behavior in selected behavioral paradigms in a sex- and protocol-specific manner (55). This suggests that further behavior tests are needed to examine whether additional mental disorders, such as schizophrenia (SCZ), are induced by the CRS protocol and DEX protocol.

Although literature on the relationship between dihydroorotate dehydrogenase (DHODH) and glucocorticoids (GCs) is limited, several studies suggest potential interactions. For example, a molecular docking study found that berberine (BBR) could stably bind to both DHODH and GC receptors (GR), indicating possible cross-talk between these proteins (56). Additionally, anti-inflammatory compounds from L. guatemalensis were shown to bind in silico to both DHODH and GRs, highlighting their shared pathway in inflammation modulation (57). High-throughput metabolic profiling has also identified DHODH inhibitors and GR agonists as key targets for regulating cancer cell metabolism, further emphasizing their relevance in cellular stress responses (58, 59). In addition to metabolic regulation, glucocorticoids (GCs) can cause neuronal atrophy and synaptic dysfunction by promoting Tau hyperphosphorylation, which disrupts cytoskeletal integrity and leads to the degradation of synaptic proteins. GCs and stress also modulate microglial activation and neuroinflammatory processes, worsening neuronal damage. Furthermore, evidence suggests that stress and GCs impact neuronal structure and function through epigenetic mechanisms (60). Thus, although the specific mechanisms connecting DHODH and GR signaling remain underexplored, existing evidence indicates that DHODH plays a role in GC-induced cellular processes, particularly in neurodegenerative pathologies. DHODH may be involved in mechanisms such as Tau hyperphosphorylation and microglial activation, offering new avenues for understanding stress-related neurodegeneration.

**Figure 6:**
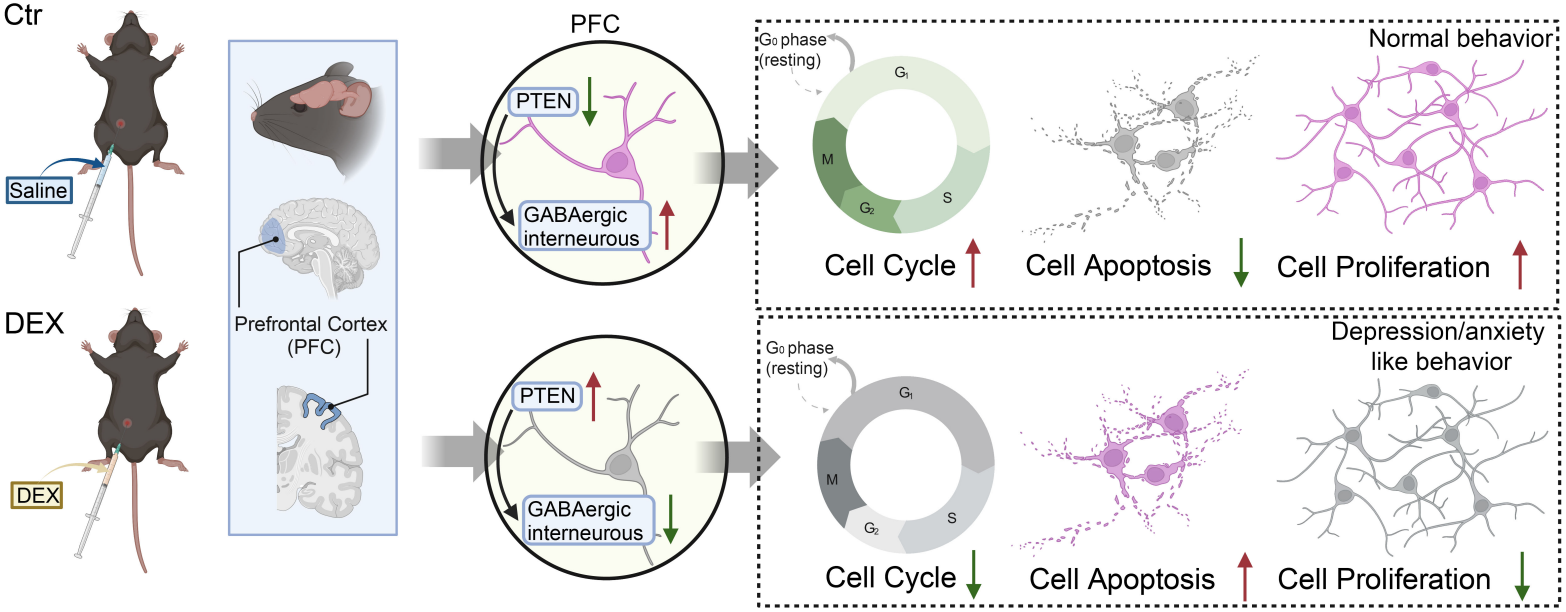
The possible mechanisms of the depression/anxiety model induced by DEX in mice, with the red arrows indicating upregulation or downregulation.

Investigations in rodent models have expanded upon these human trials and validated that similar to major depressive disorder (MDD) in humans, recurrent stress exposure and prolonged elevation of glucocorticoids in mice cause reduced body weight, reduced PFC and hippocampal volumes, and atrophy of cortical pyramidal neurons in the PFC and hippocampus (61–63), which is in accordance with our previous study. In our current research, a decrease in cortical thickness and an overall smaller brain size were noted in the DEX-treated mice. This brain atrophy might be caused by increased apoptosis of neurons and glia. Our previous data also showed that CRS and DEX treatments upregulate PTEN levels in the PFC, and the associations between PTEN genetic polymorphisms and the susceptibility to depression and symptoms of depression have been reported as well (35, 64–66). There is substantial proof supporting the significance of PTEN in tumorigenesis, where the mutation and inactivation of PTEN function can affect cellular proliferation, apoptosis, and progression through the cell cycle (67). In the developing nervous system, Pten conditional knockout (CKO) neurons show increased proliferation, cell volume and apoptosis (68). Similarly, Pten conditional deletion in astrocyte precursors leads to increased proliferation (69). In contrast, overexpression of PTEN markedly inhibits cell proliferation, promotes cell apoptosis, causes cell cycle arrest at G1 and downregulates p-AKT (67). Thus, we tentatively conclude that the cell cycle or apoptosis closely orchestrates brain development and that the control of PTEN by glucocorticoids is the bridge between them (**Figure 6**). Moreover, the next step of TUNEL staining and flow cytometry exploration in DEX-treated mice may validate our conjectures.

In the exploration of the possible mechanism underlying the behavioral alterations, we found that the DEX-treated mice presented decreased GABAergic signaling, which may be attributed to the upregulation of PTEN levels induced by DEX. Critically, the results of our evaluation show that the PV and CR types, which decreased by approximately 50%, were more susceptible than other GABAergic markers (decreased by 10%-15%) to the influence of glucocorticoids. PV interneurons represent the largest population of GABAergic neurons, constituting around 32-38% of the total GABAergic interneuron population (70), while CR interneurons constitute a heterogeneous subpopulation of approximately 10-30% of GABAergic interneurons (71). Notably, a majority of PV interneurons in the IL of the PFC express GRs, whereas GR-positive staining is not detected in other interneuron populations localized in the region (72). In adult individuals, chronic stress leads to decreased GR levels and enhanced functioning of infralimbic PV neurons (73), suggesting the possible involvement of PV neurons in disrupting the negative feedback loop of the HPA axis under chronic stress. Research demonstrated a conversion of CR-positive young neurons to GR-positive ones in the elderly population, suggesting an increase in sensitivity to corticosteroids (74). This may explain why PV and CR neurons are most susceptible to DEX.

In contemporary psychiatric studies, the disturbance of the balance of E/I has garnered significant attention (27, 75). This investigation highlights a decrease in GABAergic interneurons, signifying a potential increase in the E/I balance, resulting in amplified neuronal excitability driven by synaptic inputs. Multiple studies have documented decreased GABA concentrations in the brains of individuals with MDD over the past three decades (76), while antidepressant treatments may boost the transmission of GABAergic synapses (77). Clinical studies have demonstrated that ketamine and agmatine initially function by selectively blocking a subset of NMDA receptors on GABAergic interneurons, which leads to disinhibition of glutamatergic target neurons, a surge in extracellular glutamate accompanied by elevated glutamatergic synaptic transmission (78, 79).Our previous results suggest that the utilization of VO-OHpic, known as a PTEN inhibitor, may offer therapeutic advantages in the treatment of depression-like behaviors and PFC neuron atrophy. Thus, it was used to explore: (i) how the E/I balance is changed by VO-Ohpic, and (ii) whether VO-Ohpic is able to attenuate GABAergic disfunction and anxiety-like behaviors in Dex-treated mice. Consequently, further explicit verification will be sought in forthcoming follow-up research.

## Data Availability

The original contributions presented in the study are included in the article/**Supplementary Material**. Further inquiries can be directed to the corresponding authors.

## Supplementary material

Supplementary Figure 1 The DEX-treated mice universally presented a reduction in cortical thickness spanning from rostral to caudal at P77. (A-D) Nissl staining showed that the mice retained a six-layer cortical structure, intact hippocampal tissue, and an intact septum after DEX exposure, while the mice treated with DEX presented a reduced cortex compared with control mice at P77. Scale bars = 200μm. CC, corpus callosum; Ctx, cerebral cortex; LV, lateral ventricles; Se, septum; St, striatum; Hi, hippocampus; S1FL, primary somatosensory cortex of forelimb.
