# Supplementary figures and images for: Characterization of GABAergic marker expression in prefrontal cortex in dexamethasone induced depression/anxiety model
Source: Front Endocrinol (Lausanne). 2024 Oct 17;15:1433026. doi: 10.3389/fendo.2024.1433026 (PMC11524930; doi:10.3389/fendo.2024.1433026)

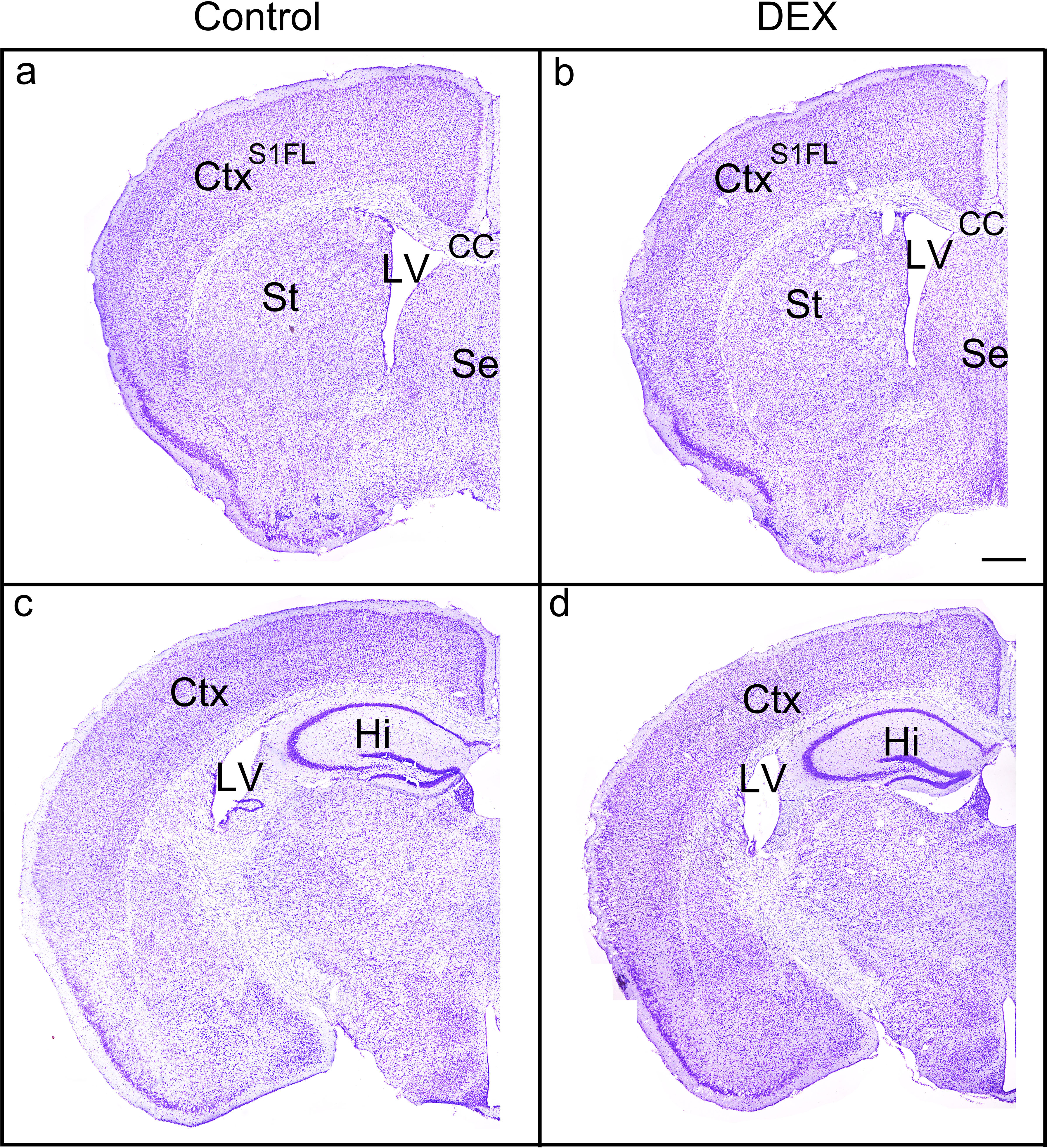

Supplement: Supplementary Figure 1 — The DEX-treated mice universally presented a reduction in cortical thickness spanning from rostral to caudal at P77. (A-D) Nissl staining showed that the mice retained a six-layer cortical structure, intact hippocampal tissue, and an intact septum after DEX exposure, while the mice treated with DEX presented a reduced cortex compared with control mice at P77. Scale bars = 200μm. CC, corpus callosum; Ctx, cerebral cortex; LV, lateral ventricles; Se, septum; St, striatum; Hi, hippocampus; S1FL, primary somatosensory cortex of forelimb. [file Image1.jpeg]
